# ALK Inhibitors in the Treatment of ALK Positive NSCLC

**DOI:** 10.3389/fonc.2018.00557

**Published:** 2019-01-09

**Authors:** Muhammad Khan, Jie Lin, Guixiang Liao, Yunhong Tian, Yingying Liang, Rong Li, Mengzhong Liu, Yawei Yuan

**Affiliations:** ^1^Department of Radiation Oncology, Affiliated Cancer Hospital and Institute of Guangzhou Medical University, Guangzhou, China; ^2^Department of Oncology, First affiliated Hospital of Anhui Medical University, Hefei, China; ^3^Department of Radiation Oncology, Sun Yat-sen University Cancer Center, Sun Yat-sen Medical University, Guangzhou, China

**Keywords:** anaplastic lymphoma kinase (ALK), non-small cell lung cancer (NSCLC), molecular targeted agents, chemotherapy, progression free survival (PFS), quality of life (Qol)

## Abstract

**Background:** ALK inhibitors have shown positive advance in the treatment of ALK+ NSCLC. They have achieved better results in prolonging the progression free survival and improving quality of life in comparison to chemotherapy. We have assembled the evidence related to the efficacy and safety of these agents in the treatment of ALK positive NSCLC.

**Materials and Methods:** A comprehensive search was conducted using electronic databases of PubMed, Medline and Cochrane Library to identify the studies involving comparison of ALK inhibitors to chemotherapy and Next generation ALK inhibitors to crizotinib. PFS was the primary outcome while other outcomes like ORR, adverse events, quality of life and OS were also analyzed and compared. Hazard ratios and odds ratios obtained were analyzed using fixed effect or random effects model in Review Manager Software.

**Results:** A total of 12 studies (*n* = 3,297) met the criteria for inclusion in this review and meta-analysis. ALK inhibitors including crizotinib, ceritinib and alectinib revealed significantly better PFS (HR 0.42 [0.35, 0.50; *p* < 0.00001]), ORR (Overall OR 6.59 [4.86, 8.94; *p* < 0.00001] as compared to chemotherapy in the first line as well as second line treatment settings. Intracranial response rate was better with ALK inhibitors (ceritinib and alectinib) as compared to chemotherapy OR 6.51 [2.86, 14.83; *p* < 0.00001]. No significant increase in grade 3 or 4 adverse events was observed with crizotinib (OR 1.21 [0.82, 1.77; *p* = *0.34*]) or ceritinib (OR 1.49 [0.86, 2.57; *p* = *0.17*]) when compared to chemotherapy individually. Quality of life indicators assessed were significantly improved with ALK inhibitors. Next generation agents (ceritinib, alectinib and brigatinib) revealed significant improvement in PFS (HR 0.50 [0.43, 0.57; *p* < 0.00001]), ORR (OR 1.57 [1.21, 2.04; *p* = 0.0006]) in comparison to crizotinib. Next generation agents (Alectinib and brigatinib) yielded better response intra-cranially than crizotinib in terms of objective response rate (OR 5.87 [3.49, 9.87; *p* < 0.00001]) and time to CNS progression (HR 0.25 [0.13, 0.46; *p* < 0.0001]). Alectinib by far resulted in fewer adverse events than chemotherapy or crizotinib.

**Conclusions:** Overall ALK inhibitors are safe and effective treatment option in ALK+ non-small cell lung cancer. Of the ALK inhibitors, Next generation agents in particular alectinib and brigatinib are safer and more effective intra-cranially and can be preferred as first option.

## Introduction

Lung cancer is the cause of 1.5 million deaths every year with < 20% of 5 year-OS for newly diagnosed patients (1). Based upon the microscopic appearance of tumor cells, lung cancers are classified into two main types: small cell lung cancer (15–20%) and non-small cell Lung cancer (80–85%) (2). NSCLC are further subdivided into three main types: adenocarcinoma (50%), squamous cell carcinomas (30%) and large cell carcinomas. This classification is based upon the types of cells found in the tumor (3). Molecular and biological targets involved in cancer growth and survival (gene mutations, proteins and signaling pathways) have been identified with progress being made in the understanding of tumor biology (4). Gene mutations like EGFR gene mutation (10–15% nsclc), KRAS mutations (10–15% nsclc) and ALK gene rearrangement (5% nsclc); Proteins like Epidermal growth factor receptor (EGFR), abnormal ALK protein; are some of the targets in NSCLC that essentially have modernized the concept of personalized medicine (5). These newer developments have essentially lead to modern molecular classification of NSCLC particularly the histology cell type adenocarcinoma.

ALK gene alterations have been well reported to play a key role in the pathogenesis of several cancers (inflammatory myofibroblastic tumors and neuroblastomas) after it was first discovered in anaplastic large cell lymphoma and hence the name anaplastic lymphoma kinase (6, 7). In 2007, ALK gene rearrangement was discovered in NSCLC: for the first time in solid tumors (8, 9). This gene alteration resulted from inter-chromosomal inversions within the short arm of chromosome 2 [Inv(2)(p21p23)] joining the exons 1–13 of the echinoderm microtubule-associated protein-like 4 (*EML4*) gene to exons 20–29 of ALK gene. The resulting EML4-ALK protein, novel to NSCLC, contains N-terminal portion encoded by (*EML4*) gene and a C-terminal portion (intracellular signaling portion of the receptor tyrosine kinase) encoded by (*ALK*) gene (10).

Anaplastic lymphoma kinase gene rearrangement is present in 3–5% of the NSCLC. Clinical features associated with this distinct NSCLC subgroup included young age, non-smoking history and adenocarcinoma histology (11). Standard chemotherapy was used as the first line of therapy before EML4-ALK discovery. Upon discovery, Crizotinib, a tyrosine kinase inhibitor targeting MET, ROS1 and ALK entered phase I trial which reported 72% 6-month progression-free survival and overall response of 57% in ALK positive NSCLC (12). This trial had led to conditional approval by the FDA. A phase II study of Crizotinib in ALK positive NSCLC also reported a similar encouraging positive results (ORR 53% (95% CI: 47–60) & median PFS 8.5 months (95% CI: 6.2–9.9) (13). On the other hand, standard single agent chemotherapies generally have produced PFS of merely 2–3 months and a 10% response rates. Consequently, a phase III study reported comparative results of Crizotinib to single agent chemotherapy (premetexed or docetaxel) in previously treated NSCLC patients with former being significantly superior in median PFS (7.7–3.0 months; *P* < 0.001) and ORR (65–20%; *P* < 0.001) (14). Crizotinib has shown significant PFS and ORR in a phase III study compared to pemetrexed plus platinum chemotherapy in the first line setting as well (15).

Crizotinib however is reported with issues of resistance and relapse owing ALK dependent and independent mechanisms (16). Ceritinib, another oral ATP-competitive, a second-generation ALK tyrosine kinase inhibitor, is similar in action to Crizotinib without MET inhibiting ability. Initially indicated on progression of disease or resistance after Crizotinib use (17, 18). However, lately Ceritinib has been compared to chemotherapy and proven superior in first line as well as second line setting (19, 20). Alectinib, yet another ALK inhibitor, is a more potent ALK inhibitor with proven activity against crizotinib resistance ALK mutations. One important aspect of alectinib is its penetration of CNS. Alectinib has also shown its superiority over chemotherapy and crizotinib in recently concluded studies (21–23). A recently concluded study has also added brigatinib as a potential ALK inhibitor option to the list (24). Ceritinib, alectinib and brigatinib are termed as Next-generation agents. Newer agents are being developed which will ever expand the treatment options for ALK positive NSCLC.

The aim of this study was to assemble the available evidence of ALK inhibitors' efficacy and safety in the treatment of ALK positive NSCLC in order to provide clinicians and practitioners a better clinical picture. As well as, review and elaborate various therapeutic aspects of these agents.

## Results

**Figure 1:**
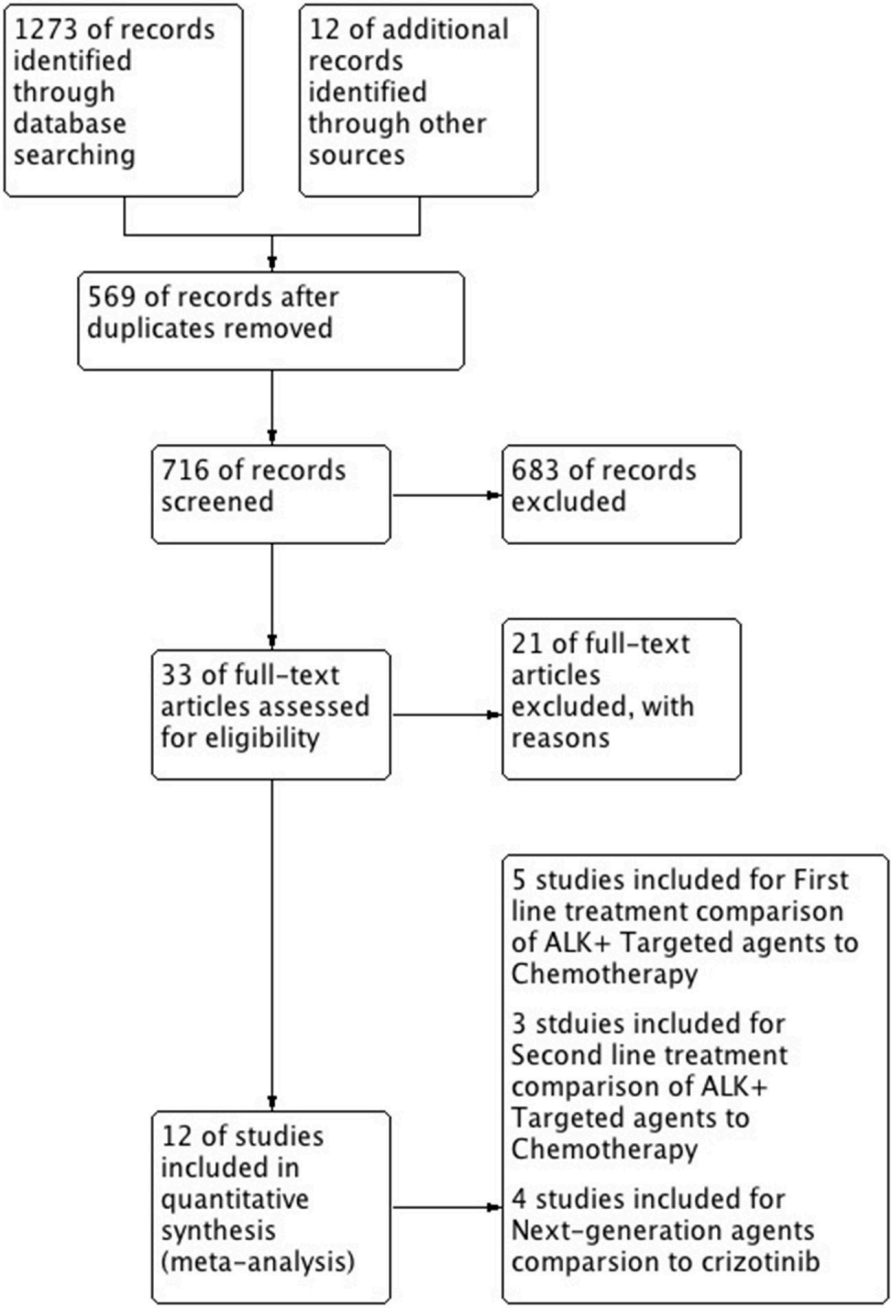
The flow diagram of literature search and selection process.

A total of 12 studies were included in this review and meta-analysis involving 3,297 patients (14, 15, 19–28). Results of research strategy and study selection is shown in Figure 1. First line comparison of ALK inhibitors vs. chemotherapy was based on 5 studies (*n* = 1,079, 4 studies included crizotinib/1 study of ceritinib vs. chemo) (15, 19, 25–27) while second line comparison was based on 3 studies (*n* = 687) (14, 20, 21). Furthermore, next generation agents (ceritinib, alectinib and brigatinib) were compared to crizotinib. Comparison was based on 4 studies involving 1,531 patients. One study was included for comparison of ceritinib to crizotinib (*n* = 746, a comparative study based on the data taken from five RCTs) (28). Two phase-III trials were identified for alectinib comparison to crizotinib and one study for comparison of brigatinib to crizotinib (22–24).

**Figure 2:**
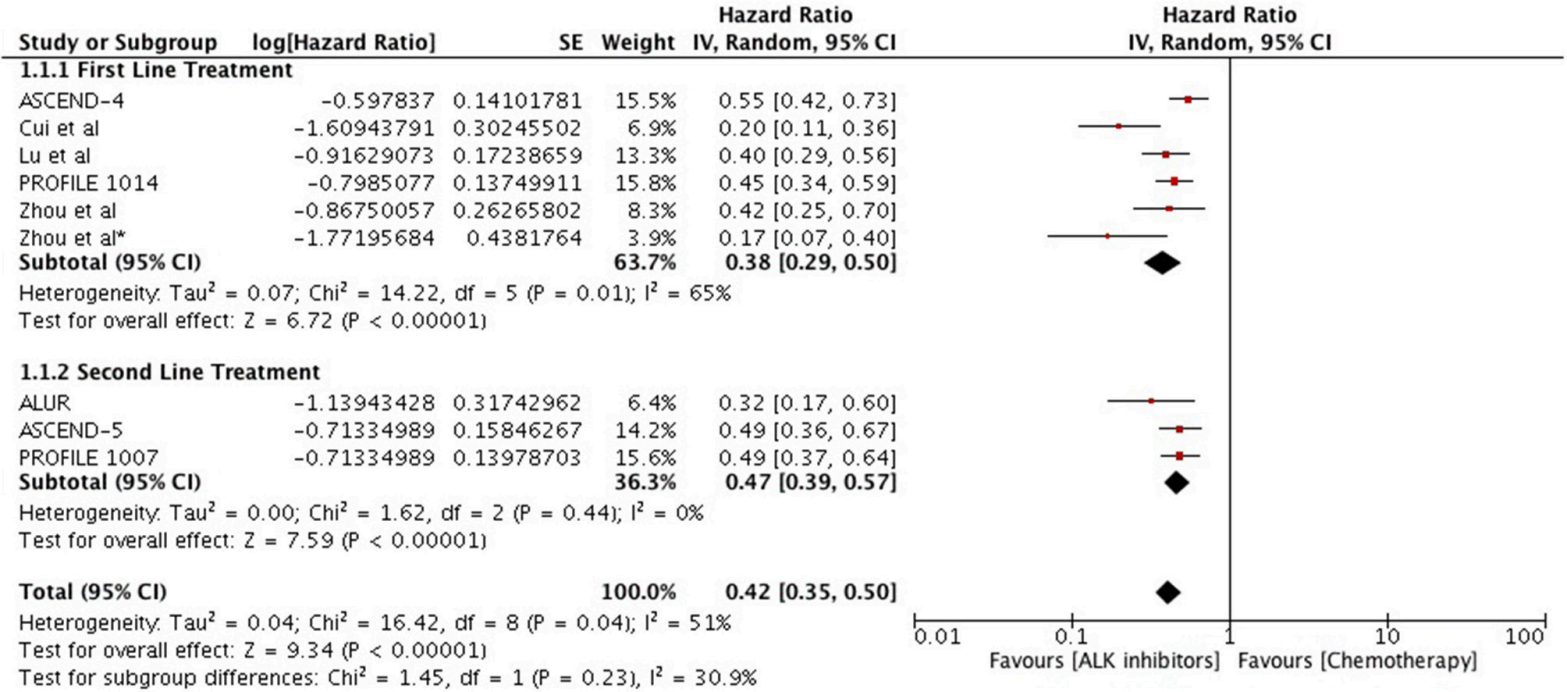
Forest plot of meta-analysis of the progression-free survival (PFS) showing comparison of ALK inhibitors to chemotherapy in ALK positive NSCLC.

**Figure 3:**
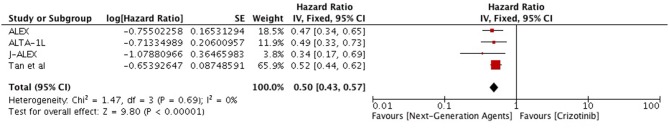
Forest plot of meta-analysis of the progression-free survival (PFS) showing comparison of Next generation agents to crizotinib in ALK positive NSCLC.

Overall, highly significant PFS was achieved with ALK inhibitors compared to chemotherapy (HR 0.42 [0.35, 0.50; *p* < 0.00001]) (Figure 2). Significant PFS with ALK inhibitors in the first line setting was based on 5 studies comprising 3 phase III trials and 2 retrospective studies. The Progression-free survival hazards ratio for ALK inhibitors (crizotinib 4 + ceritinib 1) to chemotherapy was 0.38 [0.29, 0.50; *p* < 0.00001]. Meta-analysis of ALK inhibitors in the second line setting also revealed significant PFS based on 3 phase III trials (crizotinib 1+ ceritinib1+ alectinib 1). Hazard to progression was 0.47 [0.39, 0.57; *p* < 0.00001]. Significant heterogeneity was observed and hence randome effects model was applied. Patients derived better progression free survival with next generation agents in comparison to crizotinib (HR 0.50 [0.43, 0.57; *p* < 0.00001]) (Figure 3). No heterogeneity was revealed among the studies.

**Figure 4:**
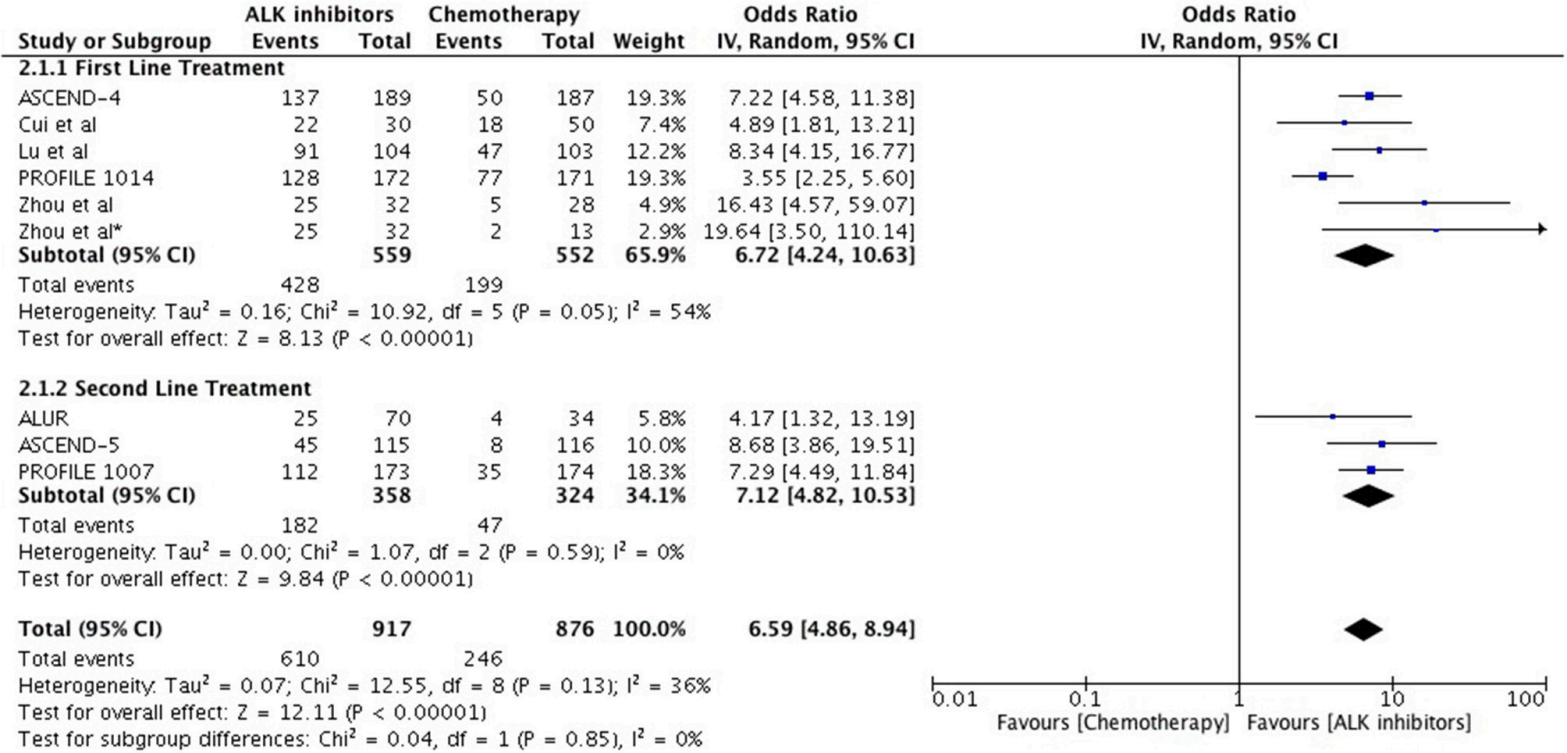
Forest plot of meta-analysis of the objective response rate (ORR) showing comparison of ALK inhibitors to chemotherapy in ALK positive NSCLC.

**Figure 5:**
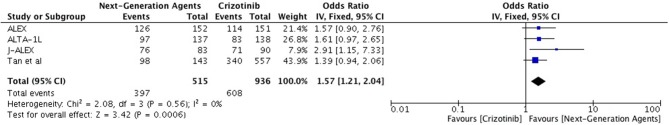
Forest plot of meta-analysis of the objective response rate (ORR) showing comparison of Next generation agents to crizotinib in ALK positive NSCLC.

Objective response was higher as well with ALK inhibitors in the first line as well as second line treatment in comparison to chemotherapy (Overall OR 6.59 [4.86, 8.94; *p* < 0.00001] (Figure 4) with no significant heterogeneity. As first line treatment, odds of achieving objective response with ALK inhibitors were significantly higher (OR 6.72 [4.24, 10.63; *p* < 0.00001]. Results are based on data from 5 studies. A similar odds ratio was observed in the second line treatment comparison (OR 7.12 [4.82, 10.53; *p* < 0.00001]. Odds of achieving objective response were significantly higher with next generation agents compared to crizotinib (OR 1.57 [1.21, 2.04; *p* = 0.0006]) (Figure 5) with heterogeneity at 0%.

**Figure 6:**
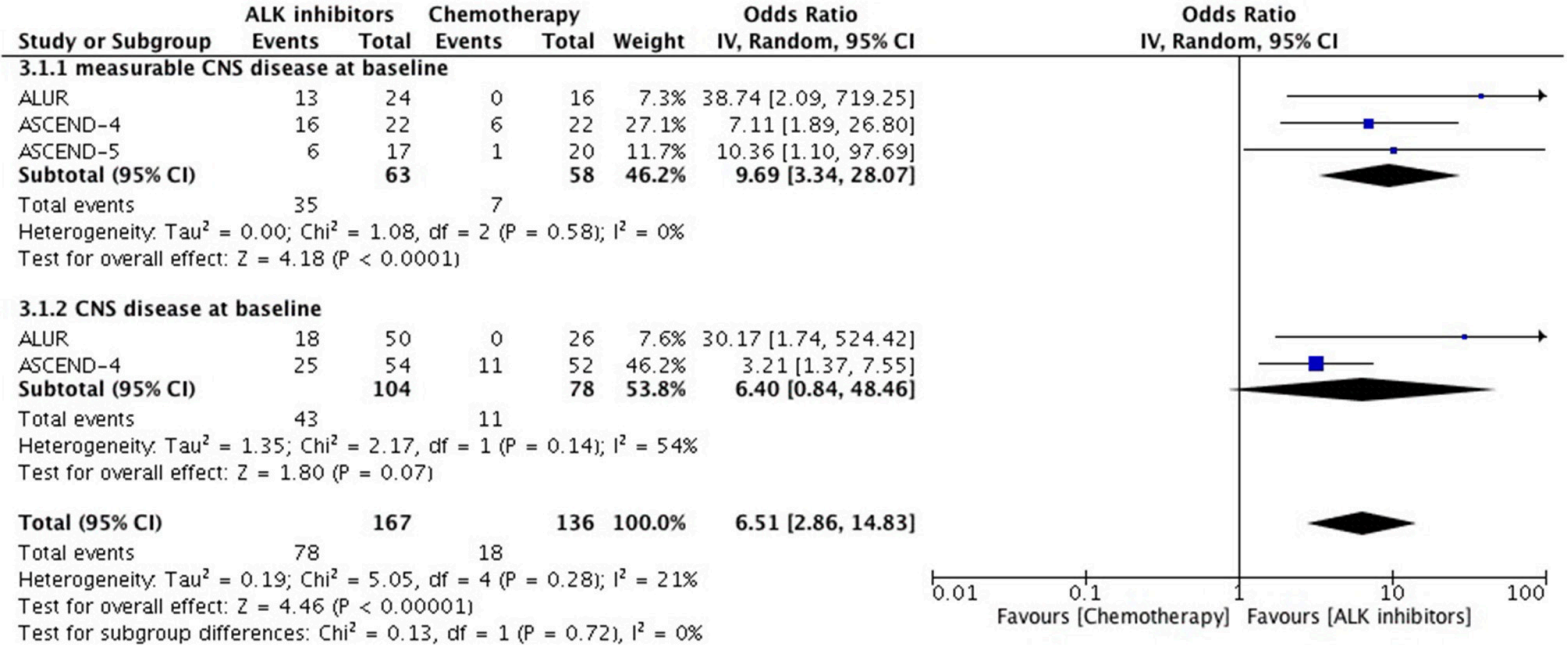
Forest plot of meta-analysis of the Intra-cranial response rate (ICRR) showing comparison of ALK inhibitors to chemotherapy in ALK positive NSCLC.

**Figure 7:**
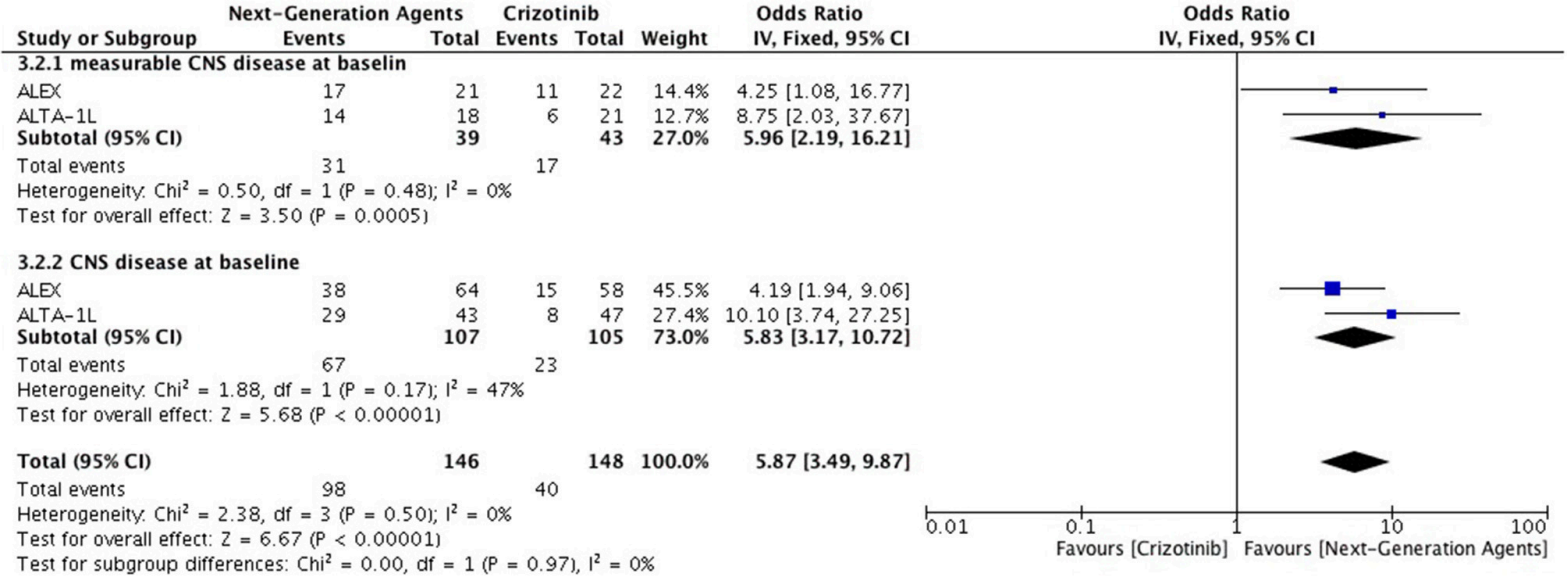
Forest plot of meta-analysis of the Intra-cranial response rate (ICRR) showing comparison of Next generation agents to crizotinib in ALK positive NSCLC.

**Figure 8:**
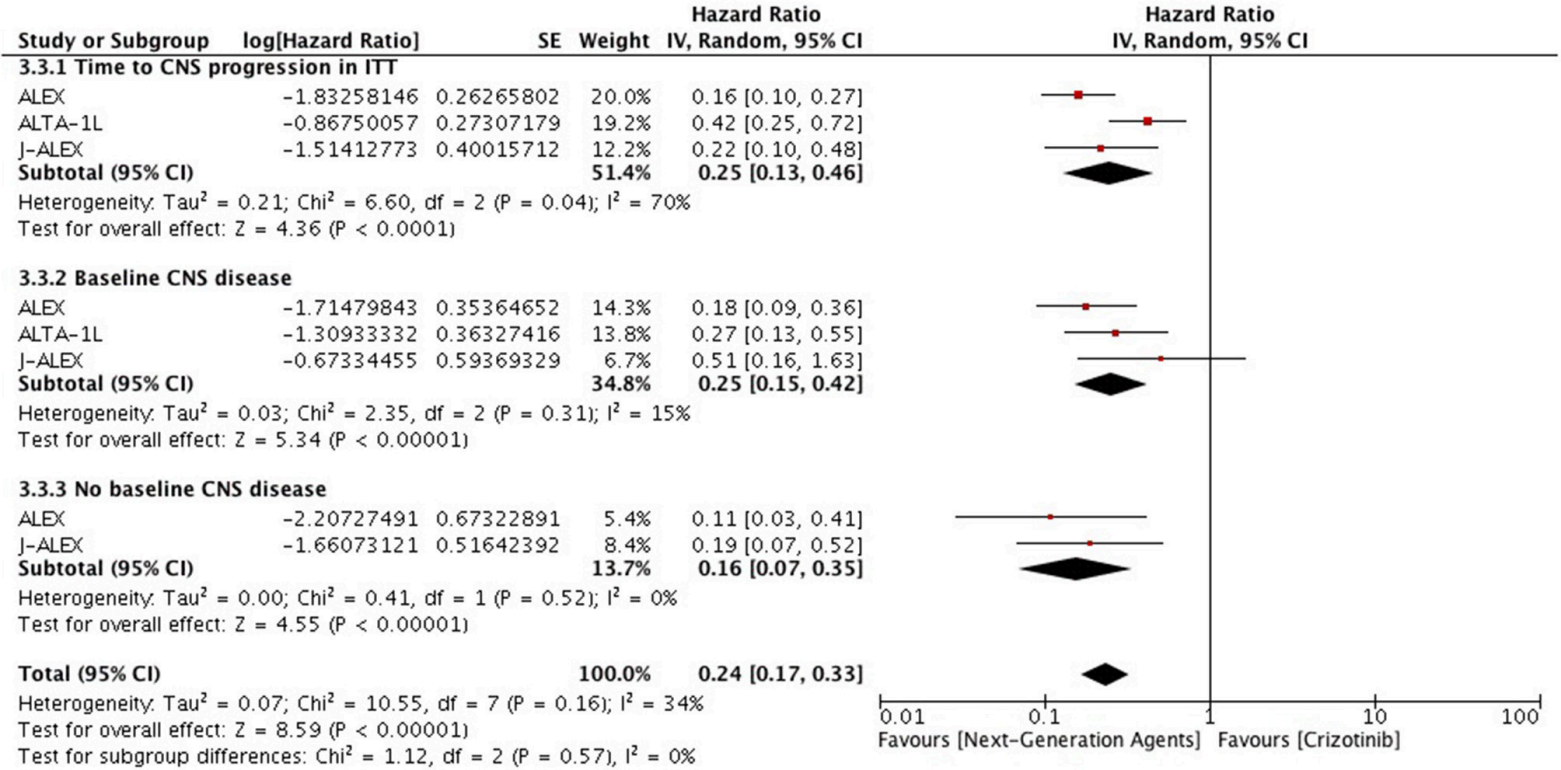
Forest plot of meta-analysis of the Time to CNS progression showing comparison of Next generation agents to crizotinib in ALK positive NSCLC.

Overall intracranial response was mainly obtained from studies comprising ceritinib and alectinib to chemotherapy. Patients responded better to ALK inhibitors in comparison to chemotherapy. A significant odds ratio was achieved without any heterogeneity among the studies (OR 6.51 [2.86, 14.83; *p* < 0.00001] (Figure 6). Indicators of response in CNS were in favor of alectinib and brigatinib in comparison to crizotinib. Odds of achieving intracranial response was significantly higher with these two agents (OR 5.87 [3.49, 9.87; *p* < 0.00001]) (Figure 7). HR for CNS progression was HR 0.25 [0.13, 0.46; *p* < 0.0001] for treatment difference in intention-to-treat population (Figure 8). Time to progression of brain metastatic lesion for patients with CNS disease at baseline was shorter (HR 0.25 [0.15, 0.42; *p* < 0.00001]) in comparison to no baseline CNS disease (HR 0.16 [0.07, 0.33; *p* < 0.00001]).

**Figure 9:**
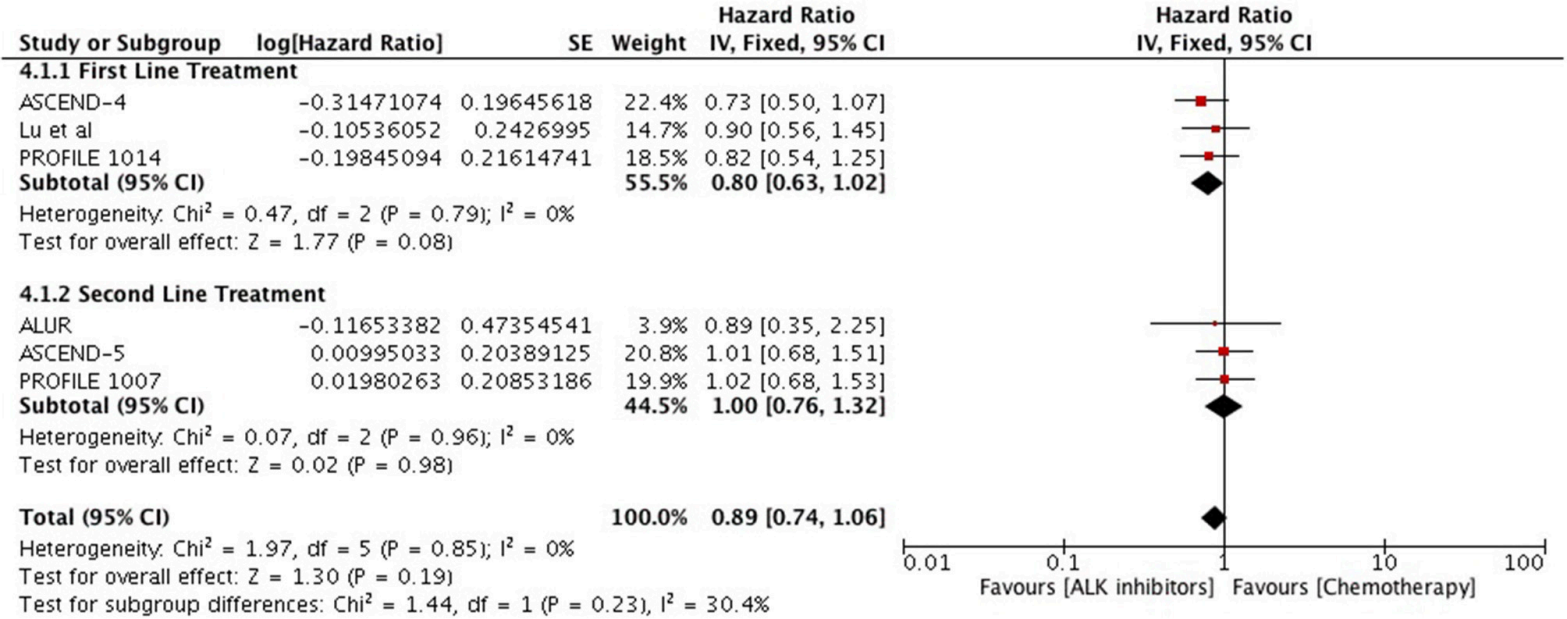
Forest plot of meta-analysis of the overall survival (OS) showing comparison of ALK inhibitors to chemotherapy in ALK positive NSCLC.

**Figure 10:**
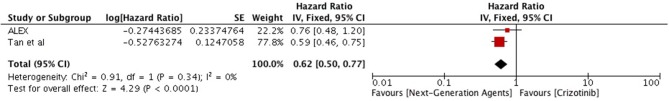
Forest plot of meta-analysis of the overall survival (OS) showing comparison of Next generation agents to crizotinib in ALK positive NSCLC.

Overall survival analysis revealed only numerical advantage over chemotherapy (HR 0.89 [0.74, 1.32; *p* = *0.19*]) particularly in first line comparison (HR 0.80 [0.63, 1.02; *p* = *0.08*] (Figure 9). Next generation ALK inhibitors revealed significant improvement in overall survival, however, result was based only on two studies (HR 0.62 [0.50, 0.77; *p* < 0.0001]) (Figure 10).

**Figure 11:**
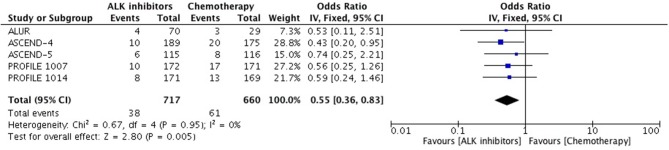
Forest plot of meta-analysis of discontinuation due to adverse events showing comparison of ALK inhibitors to chemotherapy in ALK positive NSCLC.

**Figure 12:**
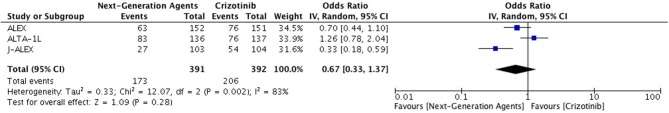
Forest plot of meta-analysis of the Grade 3 or 4 adverse events (AEs) showing comparison of Next generation agents to crizotinib in ALK positive NSCLC.

Patients receiving ALK inhibitors reported significant increase in adverse events of any grade (HR 1.63 [1.30, 2.03; *p* < 0.0001]) as well as grade 3 or 4 adverse events (HR 1.42 [1.02, 1.99; *p* = *0.04*]) in comparison to chemotherapy. Significant difference was maintained even when analyses were restricted to single agent alone. Crizotinib as well as ceritinib had lead to significant increase in causing any grade adverse events individually (Table 2). Crizotinib reported a hazard ratio of 1.52 [1.11, 2.08; *p* = *0.008*] while ceritinib reported a HR 2.09 [1.51, 2.91; *p* < 0.0001]. However, there was no difference in the crizotinib and ceritinib in comparison to chemotherapy when only grade 3 or 4 adverse events were considered for analysis (Table 3). Alectinib, on the other hand, was associated with least adverse events and caused comparatively less grade 3 or 4 adverse events compared to chemotherapy. Discontinuation of therapy from adverse events was significantly higher with chemotherapy (HR 0.55 [0.36, 0.83; *p*<*0.005*]) (Figure 11). Next generation agents mainly alectinib and brigatinib have shown overall reduction in frequency of any as well as grade 3 or 4 adverse events. However, there was no significant difference for the treatment difference in causing adverse events (Figure 12).

Quality of life was assessed in 4 studies comprising 2 studies (14, 15) comparing crizotinib and 2 studies comparing ceritinib to chemotherapy (19, 20). QLQ-C30, QLQ-C13 and EuroQol (ED-5D-5L) questionnaires were used to assess the quality of life. Global quality of life was significantly improved with crizotinib and ceritinib (*p* < 0.001). Functioning outcomes were also improved specifically physical, social and role functioning across studies. Response to QLQ-C30 Symptoms questionnaire revealed significant decrease in symptoms relief with crizotinib particularly in first line setting and ceritinib in both treatment lines as compared to chemotherapy (Table 4). These symptoms included fatigue, pain, dyspnea, insomnia and appetite loss. Significant reduction in symptoms such as dyspnea, cough, alopecia, chest pain and pain in other parts as per response to QLQ-C13 questionnaire were also reported in all the 4 studies.

**Figure 13:**
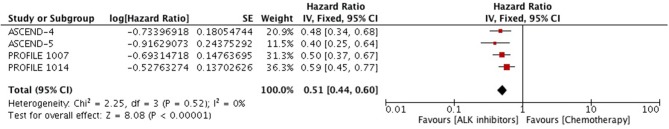
Forest plot of meta-analysis of the time to deterioration with respect to a composite end point of three symptoms–cough, dyspnea or chest pain showing comparison of ALK inhibitors to chemotherapy in ALK positive NSCLC.

Time to deterioration for the composite endpoint of Lung Cancer Specific Symptoms (LCSS) was significantly prolonged with crizotinib and ceritinib in comparison to chemotherapy. Meta-analysis of the hazard ratios revealed significant delay in time to deterioration (HR 0.51 [0.44, 0.60; *p* < 0.00001]) (Figure 13).

## Discussion

Overall these agents had been successful in controlling the progression of the disease as compared to chemotherapy. Progression free survival had been significantly improved with crizotinib and next generation ALK inhibitors as compared to chemotherapy in first line as well as second line treatment setting. Moreover, each drug individually had been proven to be effective in its comparison to chemotherapy in terms of PFS. Objective response rate as defined by combination of complete response and partial response was reported across all studies favoring targeted therapeutic agents. Patients responded in significantly high number to targeted therapies. All the studies were consistent in their results in terms of PFS and ORR favoring targeted agents. Next generation agents including ceritinib, alectinib and brigatinib were superior in terms of PFS and ORR to crizotinib.

Brain metastases are common with ALK+ NSCLC substantiating patients' symptoms (fatigue, headaches and depression), treatment cost, outpatient visits and inpatient stays (29). Chemotherapy as well as crizotinib is limited in their ability to penetrate CNS (30, 31) and hence in majority of the cases disease progression site is CNS particularly when baseline brain metastases are present which is deemed as worst prognostic factor (27). A similar intracranial response was reported in PROFILE 1,014 trial between the treatment groups (15% each) (15). However, Solomon et al. reported a non-significant improvement in Intra-cranial time to progression (HR, 0.45; *P* = 0.063) and significant improvement in intra-cranial disease control rate with crizotinib at 12 weeks (85 vs. 45%, respectively; *P* < 0.001) and at 24 weeks (56 vs. 25%, respectively; *P* = 0.006) as compared to chemotherapy in a population of stable treated

brain metastases (32). In contrast, next generation inhibitors, ceritinib and alectinib, has been more potential CNS penetrant (33). Our meta-analysis showed a significantly better intra-cranial response with ceritinib and alectinib in comparison to chemotherapy. Next generation agents have shown a greater potency in the brain overall. Alectinib and brigatinib have reported significantly better response intra-cranially. These agents also have significantly delayed CNS disease progression in patients with or without baseline CNS disease as well as with or without prior radiotherapy which has been associated with better response from targeted therapy (34, 35).

Survival analysis must be interpreted carefully as either the data was immature or high crossover was reported. PROFILE 1,014 reported no significant survival difference with about 70% crossover from chemotherapy to crizotinib group. Rank-preserving structural failure time model was used for adjusting the cross-over which revealed a better overall survival with crizotinib as calculated with Wilcoxon test (HR 0.60 [0.27, 1.42; *p* < 0.00001]) and log-rank test (HR 0.67 [0.28, 1.48; *p* < 0.00001]). This outcome suggested that cross over might have confounded the overall survival analysis (15). Final OS analysis of PROFILE 1,014 reported better OS achieved with crizotinib after adjustment for crossover (HR 0.346 [0.081, 0.718]) (36). Lu et al. survival analysis was based only on 35% of OS events while 82 (80%) patients had crossed over to crizotinib (26). Despite such a huge crossover in first line comparison, a numerical advantage close to being statistically significant was yet achieved. A similar trend of crossover has also been reported in each individual study comprising second line treatment comparison (14, 20, 21). The improvement in progression free survival might have been resulted in significant OS given the maturity of the study was reached and high crossover was prevented. Duruisseaux et al. (37) reported a better median OS of 16.6 months with crizotinib in unselected ALK+ NSCLC patients. It also reported that the line of treatment was not associated with survival outcome.

ALK inhibitors (except for alectinib) had been shown to cause significantly higher number of grade 1 and 2 adverse events. Vision disorders, diarrhea, edema, vomiting, elevated aminotransferases, cough, back pain, upper abdominal pain, weight decrease, blood alkaline phosphatase increase, blood creatinine increase, gamma-glutamyltransferase (GGT) increase and non-cardiac chest pain were reported predominantly in ALK inhibitors group. While patients receiving chemotherapy reported fatigue, alopecia, anemia, neutropenia, leukopenia. A number of adverse events were reported similar in frequency in both treatment groups. These included cough, nausea, dizziness, dyspnea, constipation, decreased appetite, asthenia and rash. Several adverse events were unique to each agent individually. Vision disorders, dizziness, dysgeusia and edema were only reported with crizotinib. Ceritinib also reported a number of distinct adverse events such as back pain, upper abdominal pain, weight decrease, blood alkaline phosphatase increase, blood creatinine increase, gamma-glutamyltransferase (GGT) increase and non-cardiac chest pain. Analysis was not adjusted for the duration of treatment as duration of crizotinib treatment was comparatively longer as compared to chemotherapy. Median duration of treatment was 10.6 months and 31 weeks in crizotinib group compared to 4.1 months and 21 weeks in chemotherapy group in the PROFILE 1,014 and 1,007 trial, respectively. A similar longer duration was also reported with ceritinib as well.

Grade 3 and 4 adverse events were comparatively similar in frequency in both treatments. There was no difference between the treatments when each single agent (ceritinib or crizotinib) was compared to chemotherapy in causing grade 3 or 4 adverse events. Total 6 patients receiving crizotinib qualified for Hy's law criteria leading to discontinuation of treatment (5 patients were reported in PROFILE 1,014 and 1 from PROFILE 1,007). Three of the 6 patients were with grade 2 or 3 elevated aminotransferases and one with drug-induced hepatic injury. No patient was reported in ceritinib group meeting Hy's criteria. Overall adverse events leading to permanent discontinuation of treatment were comparatively higher in chemotherapy group compared to crizotinib and ceritinib. Most of the adverse events were managed with dose adjustments, interruptions or delays.

Quality of life was assessed with changes from baseline on the European Organization for Research and Treatment of Cancer (EORTC) quality of life core questionnaire (QLQ-C30), module for lung cancer (QLQ-LC13) and EuroQol Group 5-Dimension Self-Report Questionnaire (EQ-5D) scales. Ceritinib showed a decrease in peripheral neuropathy while crizotinib was associated with an increase. Ceritinib also lead to significant decrease in symptoms like sore mouth and dysphagia. Pain in arm or shoulder was significantly reduced with crizotinib therapy and no significant difference was reported with ceritinib in comparison to chemotherapy. Overall a first line use reported better control of these agents in symptoms reduction and improvement of quality of life as compared to second line treatment setting.

There are several limitations to this study. Not all the studies included were of high quality. Phase III trials were not blinded. Two of the studies included were of retrospective nature with high chance of incurring selection bias (25, 27). Results of one study comparing ceritinib to crizotinib based on data derived from 5 different trials involving the two agents could be confounding due to non-randomization, study level data incorporation and inherent limitations of the involved studies (28). A high crossover has been reported with all the 6 studies comprising survival analysis and might have confounded the survival outcome. In PROFILE 1014, maintenance therapy with premetexed was not continued after initially planned six cycles of premetexed-plus-platinum, which might have affected the progression free survival in chemotherapy group as reported in PARAMOUNT study, however, ever so slightly (38).

Main aim of the study was to highlight the progress being made in ALK inhibitors treatment of ALK positive NSCLC. Since a number of agents being approved with next-generation agents more efficacious in CNS metastatic disease, it is debatable to choose an agent as initial choice. Crizotinib is usually given as first line treatment choice. Relapse is more common with crizotinib. Both next generation agents have been proven efficacious in crizotinib resistant disease (35, 36). On other hand, ceritinib and particularly alectinib is more efficacious in controlling the progression of the disease compared to crizotinib and more active in CNS metastatic lesions which is a main progression site in crizotinib treated patients. Alectinib has a strong safety profile when compared to crizotinib and ceritinib. Newer agents include brigatinib, lorlatinib, ensartinib and entrectinib are making their way to enter the treatment paradigm of ALK+ NSCLC. Brigatinib has already been approved and lorlatinib has also shown good efficacy (39, 40). The spot for first choice of treatment dimension is being changed over time with the addition of brigatinib showing comparability to alectinib in all aspects of treatment efficacy and safety (24). Currently a clinical trial (NCT03596866) is undergoing comparing brigatinib to alectinib in advanced ALK positive NSCLC patients who have progressed on crizotinib (41). Ensartinib and entrectinib are under investigational stages.

## Conclusions

ALK inhibitors collectively and individually have shown significant improvement in PFS, ORR, Quality of life without any increase in toxicity (grade 3,4 adverse events) compared to chemotherapy. First line as well as second line treatment comparison revealed a similar prominent picture of ALK inhibitors' efficacy. ALK inhibitors clearly represent a better choice of treatment and could be recommended and preferred over chemotherapy. Alectinib has shown all positive indicators to be first choice of ALK positive NSCLC treatment as it has reported its superiority over chemotherapy as well as crizotinib in terms of PFS, ORR, and intracranial efficacy and by far safer to other agents including ceritinib. Recently, alectinib is being compared to brigatinib for superiority. Dimension of ALK positive NSCLC treatment is undergoing fast development with the addition of newer ALK inhibitors.

## Materials and Methods

### Search Strategy and Study Selection

PubMed, Medline and Cochrane Library were searched comprehensively until Sep 2018 using a wide range of terms including “ALK+ NSCLC” OR “ALK positive non-small cell lung cancer” AND “ALK inhibitors” OR “ALK” OR “crizotinib” OR “ceritinib” OR “alectinib” OR “brigatinib” OR “Next generation ALK inhibitors” AND “Chemotherapy.” Titles and abstracts of the retrieved studies were screened for eligibility. Full texts screening was done to include studies qualifying for inclusion according to eligibility criteria.

### Eligibility Criteria

Published in English studies comparing the “ALK inhibitors with chemotherapy” and “next generation inhibitors to crizotinib” in the treatment of ALK+ non-small cell lung cancer. Comparisons of interests were: crizotinib vs. chemotherapy; ceritinib vs. chemotherapy; alectinib vs. chemotherapy; ceritinib vs. crizotinib and alectinib vs. crizotinib.

### Outcomes of Interest and Data Extraction

Progression-free survival was the primary outcome of interest. Secondary outcome of interests included objective response rate, overall survival, intracranial efficacy, adverse events and quality of life. Data was extracted from all the studies included general characteristics of the trial, patient's characteristics and main outcomes of interest (Table 1).

**Table 1:** General characteristics of the studies and participants.

**Characteristics**	**1st Line Treatment**					**2nd Line Treatment**			**Next generation**			
Title	PROFILE 1014	Cui et al	Lu et al	Zhou et al	ASCEND-4	PROFILE 1007	ASCEND-5	ALUR	Tan et al	ALEX	J-ALEX	ALTA-1L
Year	2013	2016	2016	2018	2017	2014	2017	2017	2016	2017	2017	2018
Design	Phase III	Retrospective	Phase III	Retrospective	Phase III	Phase III	Phase III	Phase III	Retrospective	Phase III	Phase III	Phase III
No. of Patients	343:172/171	80:30/50	207:104/103	73:32/28/13	376:189/187	347:173/174	231:115/116	109:72/35	709:189/557	303:152/151	207:103/104	275:137/138
Age: Exp Con	52(22–76) 54(19–78)	58(37–83) 52(26–72)	NA		55(22–81) 54(22–80)	51(22–81) 49(24–85)	54(44–63) 54(47–64)	55.5(21, 82) 59.0(37, 80)	52 52	54(18–91) 58(25–88)	61(27–85) 59(25–84)	58(27–86) 60(29–89)
Male Female	131:68/63	38:15/23 42:15/27	NA	36:19/11/6 37:13/17/7	160:87/73 216:102/114	153:75/78	102:47/55 129:68/61	58:41/17 49:31/18	378:77/301	132:64/68 171:87/84	82:41/41 125:62/63	125:68/57 150:69/81
No of BM	45/47	NA	21/32	NA	59/62	60/60	65/69	47/26	NA	58/64	16/31	40/41
Experimental agent	Crizotinib 250 mg PO BID	Crizotinib 250 mg PO BID	Crizotinib 250 mg PO BID	Crizotinib 250 mg PO BID	Ceritinib 750 mg PO QD q3w	Crizotinib 250 mg PO BID	Ceritinib 750 mg PO QD q3w	Alectinib 600 mg PO BID	Ceritinib 750 mg PO QD q3w	Alectinib 600 mg PO BID	Alectinib 600 mg PO BID	Brigatinib 180 mg once daily with a 7-day lead-in at 90 mg
Control	Chemo: Pemetrexed 500 mg/m^2^ with either cisplatin 75 mg/m^2^ or carboplatin AUC 5–6, IV q3w for ≤ 6 cycles	Chemo: Pemetrexed 500 mg/m^2^, docetaxel 75 mg/m^2^, or gemcitabine 1,250 mg/m^2^ on days 1 and 8 with either cisplatin 75 mg/m^2^ or carboplatin AUC 5–6, IV q3w	Chemo: Pemetrexed 500 mg/m^2^ with either cisplatin 75 mg/m^2^ or carboplatin AUC 5–6, IV q3w for ≤ 6 cycles	Chemo: Platinum based Pemetrexed /Platinum based non-pemetrexed	Chemo: Pemetrexed 500 mg/m^2^ with either cisplatin 75 mg/m^2^ or carboplatin AUC 5–6 for 4 cycles followed by maintenance pemetrexed	Chemo: Pemetrexed 500 mg/m^2^ or docetaxel 75 mg/m^2^ q3w	Chemo: Pemetrexed 500 mg/m^2^ or docetaxel 75 mg/m^2^ q3w	Chemo Pemetrexed 500 mg/m^2^ or docetaxel 75 mg/m^2^ q3w	Crizotinib 250 mg PO BID	Crizotinib 250 mg PO BID	Crizotinib 250 mg PO BID	Crizotinib 250 mg PO BID
PFS Exp vs. Con	MPFS: 10.9 m vs. 7.0 m HR: 0.45(0.35–0.6) *p* < 0.001	MPFS: 13.3 m vs. 5.4 m HR: 0.20(0.11–0.36) *p* < 0.001	MPFS: 11.1 m vs. 6.8 m HR: 0.40(0.29–0.57) *p* < 0.0001	MPFS: 16.1 m vs. 6 m HR: 0.37(0.28–0.43) *p* < 0.001 ^*^MPFS: 16.1 m vs. 2.9 m HR: 0.18(0.12–0.21) *p* < 0.001	MPFS: 16.6 m vs. 8.1 m HR: 0.55(0.42–0.73) *p* < 0.00001	MPFS: 7.7 m vs. 3.0 m HR: 0.49(0.37–0.64) *p* < 0.001	MPFS: 5.4 m vs. 1.6 m HR: 0.49(0.36–0.67) *p* < 0.0001	MPFS: 7.1 m vs. 1.6 m HR: 0.32(0.17–0.59) *p* < 0.001	MPFS: 13.8 vs. 8.3 m HR: 0.52(0.44–0.62) *p* < 0.001	MPFS: not reached vs. 11.1 m HR: 0.47(0.34–0.65) *p* < 0.001	MPFS: not reached vs. 10.2 m HR: 0.34(0.17–0.71) *p* < 0.0001	MPFS: not reached vs. 9.8 m HR: 0.49(0.33–0.74) *p* < 0.001
ORR	Exp: 128/172, 74% (67–81) Con: 77/171, 45% (37–53)	Exp: 22/30, 73.3% (57–89) Con: 18/50, 36% (23–49)	Exp: 91/104, 87.5% Con: 47/103, 45.6%	Exp: 32/32, 100% Con: 1-PP:5/28, 17.9% N1-PP:2/13, 15.4%	Exp: 137/189, 72.5% (65–79) Con: 50/187, 27% (20–34)	Exp: 113/173, 65% (58–72) Con: 34/174, 20% (14–26)	Exp: 45/115, 39% (30–49) Con: 8/116, 6.9% (3–13)	Exp: 27/72, 37.5% Con: 1/35, 2.9%		Exp: 126/152, 83% (76–88) Con: 114/151, 75.5% (68–82)	Exp: 76/83, 92% (86–97) Con: 71/90, 79% (70–87)	Exp: 97/137, 71% (62–78) Con: 83/138, 60% (51–68)
IC RR	IC progression/New IC lesions Exp: 25/172, 15% Con: 26/171, 15%	NA	NA	NA	ICRR BBM = Exp: 25/54, 46.3% (33–60) Con: 11/52, 21.2% (11–35) mBBM = Exp: 16/22, 72.7% (50–89) Con: 6/22, 27.3% (11–50)	NA	NA	ICRR BBM = Exp: 18/50, 36% Con: 0/26, 0% mBBM = Exp: 13/24, 54.2% Con: 0/16, 0%		ICRR BBM = Exp: 38/64, 59% (46–71) Con: 15/58, 26% (15–39) mBBM = Exp: 17/21, 81% (58–95) Con: 11/22, 50% (28–72)		ICRR BBM = Exp: 29/43, 67% (51–81) Con: 8/47, 17% (8–31) mBBM = Exp: 14/18, 78% (52–94) Con: 6/21, 29% (11–52)
OS	HR: 0.82(0.54–1.26) *p =* 0.36	NA	HR: 0.90(0.56–1.45) *p =* 0.33	NA	HR: 0.73(0.50–1.08) *p =* 0.056	HR: 1.02(0.68–1.54) *p =* 0.54		HR: 0.89(0.35–2.24) *p = NS*	HR: 0.59(0.46–0.75) *p* < 0.001	HR: 0.76(0.48–1.20) *p =* 0.24	
Adverse events	NA	Overall AEs: l 75/80, 93.6%	NA	NA	All grades: Exp:189/189, 100% Con: 170/175, 97% Grade 3 or 4: Exp:148/189, 78% Con:108/175, 62%	NA	All grades: Exp:49/115, 43% Con: 36/113, 32%	All grades: Exp:54/70, 77% Con: 29/34, 85% Grade 3–5: Exp:19/70, 27% Con:14/34, 41%		All grades: Exp:147/152, 97% Con: 146/151, 97% Grade 3–5: Exp:63/152, 41% Con:76/151, 50%		All grades: Exp:132/136, 97% Con: 137/137, 100% Grade 3–5: Exp:83/136, 61% Con:76/137, 55%
Cross over	70%		82(80%)	NA	72%	112 (64%)		70.6%				25%

*BM, brain metastases; NA, not available; Exp, experimental group; Con, control group; Chemo, chemotherapy; PFS, progression free survival*;

* *MPFS, median progression free survival for crizotinib versus platinum based non-pemetrexed; HR, hazards ratio; ORR, objective response rate; m, months; IC, intracranial; ICRR, intracranial response rate; BBM, baseline brain metastases; mBBM, measurable baseline brain metastases; OS, overall survival; AEs, adverse events*.

**Table 2:** Any grade adverse events reported with ALK + inhibitors and chemotherapy.

**Adverse Events**	**Studies**	**No. of Part**	**Odds ratio**	**Significance**	**I^2^**
**FREQUENT WITH TARGETED THERAPY**					
Vision disorders	3	789	18.91 [12.53, 28.55]	*P* < 0.00001	0
Blood ALK increased	2	592	11.96 [3.84, 37.22]	*P* < 0.0001	32
Blood Cr increased	2	592	8.70 [0.48, 157.12]	*P =* 0.14	76 (*P =* 0.04)
GGT increased	2	592	7.17 [2.66, 19.31]	*P* < 0.0001	47
Diarrhea	6	1,485	7.14 [2.99, 17.05]	*P* < 0.00001	90 (*P* < 0.00001)
Dysgeusia	2	683	4.43 [2.36, 8.30]	*P* < 0.00001	41
Upper abdominal pain	2	592	4.19 [2.31, 7.60]	*P* < 0.00001	0
Edema	3	789	3.80 [1.72, 8.41]	*P =* 0.0010	71 (*P =* 0.03)
Elevated ALT/AST	5	2,079	3.98 [2.43, 6.53]	*P* < 0.00001	77 (*P =* 0.002)
Vomiting	5	1,381	3.46 [1.71, 6.99]	*P =* 0.0006	87 (*P* < 0.00001)
Weight decreased	2	592	3.22 [0.93, 11.10]	*P =* 0.06	83 (*P =* 0.01)
abdominal pain	3	932	3.05 [2.09, 4.45]	*P* < 0.00001	0
URTI	2	683	2.79 [1.88, 4.15]	*P* < 0.00001	0
Non-cardiac chest pain	2	592	2.65 [1.55, 4.55]	*P =* 0.0004	0
Back pain	2	592	1.87 [0.55, 6.31]	*P =* 0.31	83 (*P = 0.01*)
Dizziness	3	789	1.58 [0.63, 3.99]	*P =* 0.33	73 (*P = 0.02*)
Headache	3	932	1.47 [1.03, 2.09]	*P =* 0.03	0
Pyrexia	3	932	1.46 [1.02, 2.10]	*P =* 0.04	0
**SIMILAR FREQUENCY IN THE TWO TREATMENT GROUPS**					
Nausea	6	1,485	1.35 [0.67, 2.74]	*P =* 0.40	89 (*P* < 0.00001)
Cough	3	932	1.28 [0.90, 1.83]	*P =* 0.17	13
Constipation	6	1,485	1.26 [0.74, 2.14]	*P =* 0.39	76 (*P =* 0.0008)
Decreased appetite	4	1,038	0.96 [0.44, 2.07]	*P =* 0.92	87 (*P* < 0.0001)
Dyspnea	5	1,379	0.86 [0.64, 1.17]	*P =* 0.34	5
Asthenia	3	932	0.77 [0.44, 1.35]	*P =* 0.36	64
Rash	4	781	0.59 [0.30, 1.14]	*P =* 0.12	34
**FREQUENT WITH CHEMOTHERAPY**					
Fatigue	6	1,485	0.53 [0.31, 0.89]	*P =* 0.02	78 (*P =* 0.0003)
Leukopenia	2	704	0.28 [0.12, 0.64]	*P =* 0.003	55
Anemia	2	704	0.26 [0.17, 0.40]	*P* < 0.00001	12
Neutropenia	4	1,036	0.25 [0.11, 0.60]	*P =* 0.002	73 (*P =* 0.02)
Alopecia	4	781	0.23 [0.12, 0.43]	*P* < 0.00001	19
				
Overall	6	28,360	1.63 [1.30, 2.03]	*P* < 0.0001	92 (*P* < 0.00001)
Crizotinib vs. Chemotherapy	3	13,320	1.52 [1.11, 2.08]	*P =* 0.008	92 (*P* < 0.00001)
Ceritinib vs. Chemotherapy	2	14,208	2.09 [1.51, 2.91]	*P* < 0.0001	92 (*P* < 0.00001)

*PROFILE 1014 Trial, PROFILE 1007, ASCEND-4 listed any grade adverse events if they were reported in at least >15% patients while Cui et al and ASCENd-5listed if they were reported in at least >10% patients. Grade 3 or 4 adverse events were reported in at least >2% patients in PROFILE 1014 Trial, at least ≥3% in PROFILE 1007, at least >15% in ASCEND-4 and all grade 3 or 4 adverse events in ASCENd-5*.

**Table 3:** Grade 3 or 4 adverse events reported with ALK + inhibitors and chemotherapy.

**Adverse Events**	**Studies**	**No. of Part**	**Odds ratio**	**Significance**	***I^2^***
**FREQUENT WITH TARGETED THERAPY**					
GGT increased	2	592	24.80 [8.93, 68.86]	*P* < 0.00001	0
Blood ALP increased	2	592	14.49 [2.75, 76.47]	*P =* 0.002	0
Elevated ALT/AST	5	2,079	8.85 [4.99, 15.69]	*P* < 0.00001	28
Constipation	2	683	8.05 [1.00, 64.79]	*P =* 0.05	0
Weight decreased	2	592	4.62 [0.99, 21.71]	*P =* 0.05	0
Upper abdominal pain	2	592	4.56 [0.52, 40.40]	*P =* 0.17	0
Diarrhea	4	1,273	3.56 [1.27, 9.96]	*P =* 0.02	0
abdominal pain	2	592	2.75 [0.33, 22.79]	*P =* 0.35	9
Pneumonia	3	911	2.29 [0.93, 5.64]	*P =* 0.07	0
Non-cardiac chest pain	2	592	2.20 [0.32, 15.14]	*P =* 0.42	0
**SIMILAR FREQUENCY IN THE TWO TREATMENT GROUPS**					
Vomiting	5	1,381	1.58 [0.63, 3.96]	*P =* 0.33	38
Pulmonary embolism	3	789	1.48 [0.69, 3.19]	*P =* 0.32	8
Nausea	4	1,275	1.20 [0.38, 3.75]	*P =* 0.75	48
Decreased appetite	3	932	01.19 [0.38, 3.73]	*P =* 0.76	0
Fatigue	5	1,381	1.00 [0.55, 1.82]	*P =* 0.99	0
Headache	3	932	0.87 [0.32, 2.41]	*P =* 0.79	0
Dyspnea	4	1,275	0.77 [0.39, 1.51]	*P =* 0.45	19
Dizziness	3	911	0.45 [0.08, 2.72]	*P =* 0.39	0
Asthenia	3	932	0.73 [0.33, 1.61]	*P =* 0.43	0
Pyrexia	3	932	0.68 [0.09, 5.15]	*P =* 0.71	22
Back pain	2	592	0.55 [0.16, 1.92]	*P =* 0.35	0
**FREQUENT WITH CHEMOTHERAPY**					
Neutropenia	4	1,275	0.29 [0.10, 0.81]	*P =* 0.02	75 (*P =* 0.007*)*
Anemia	3	1,047	0.25 [0.09, 0.71]	*P =* 0.009	32
Leukopenia	3	932	0.20 [0.07, 0.62]	*P =* 0.005	0
				
Overall	6	22,856	1.42 [1.02, 1.99]	*P = 0.04*	68 (*P* < 0.00001*)*
Crizotinib vs. Chemotherapy	3	10,312	1.21 [0.82, 1.77]	*P =* 0.34	48 (*P =* 0.001*)*
Ceritinib vs. Chemotherapy	2	12,180	1.49 [0.86, 2.57]	*P =* 0.17	76 (*P* < 0.00001)

*Grade 3 or 4 adverse events were reported in at least >2% patients in PROFILE 1014 Trial, at least ≥3% in PROFILE 1007, at least >15% in ASCEND-4 and all grade 3or 4 adverse events in ASCENd-5*.

**Table 4:** Quality of life assessment reported with ALK inhibitors and chemotherapy.

	**PROFILE 1014**	**PROFILE 1007**	**ASCEND-04**	**ASCEND-05**
**GLOBAL QUALITY OF LIFE (QLQ-C30)**				
	*P* < 0.001 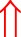	*P* < 0.001 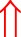	*P* < 0.001 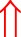	NS
**FUNCTIONING (QLQ-C30)**				
Physical	*P* < 0.001 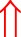		*P* < 0.001 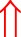	*P* < 0.05 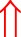
Social	*P* < 0.001 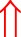		*P* < 0.05 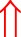	*P* < 0.05 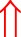
Role	*P* < 0.001 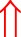		*P* < 0.001 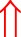	*P* < 0.05 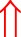
Cognitive	*P* < 0.05 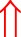		
Emotional	*P* < 0.001 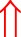		*P* < 0.05 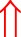
**SYMPTOMS (QLQ-C30)**				
Fatigue	*P* < 0.001 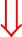	*P* < 0.001 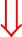	*P* < 0.001 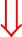	*P* < 0.05 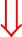
Nausea and vomiting	*P* < 0.05 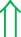		*P* < 0.001 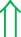	*P* < 0.05 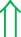
Pain	*P* < 0.001 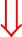		*P* < 0.05 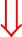
Dyspnea	*P* < 0.001 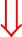		*P* < 0.001 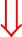	*P* < 0.001 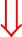
Insomnia	*P* < 0.001 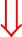		*P* < 0.05 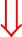
Appetite loss	*P* < 0.001 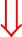		
Constipation			*P* < 0.001 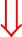
Diarrhea	*P* < 0.001 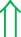		*P* < 0.001 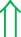	*P* < 0.001 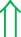
Financial difficulties			*P* < 0.05 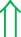	*P* < 0.05 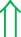
**SYMPTOMS (QLQ-LC13)**				
Dyspnea	*P* < 0.001 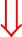	*P* < 0.001 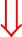	*P* < 0.001 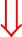	*P* < 0.001 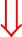
Cough	*P* < 0.001 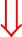	*P* < 0.001 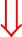	*P* < 0.001 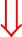	*P* < 0.001 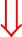
Hemoptysis			
Sore mouth			*P* < 0.001 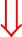	*P* < 0.001 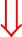
Dysphagia			*P* < 0.05 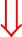	*P* < 0.05 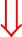
Peripheral neuropathy	*P* < 0.05 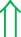		*P* < 0.001 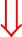	*P* < 0.001 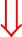
Alopecia	*P* < 0.05 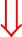	*P* < 0.05 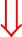	*P* < 0.001 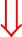	*P* < 0.001 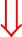
Pain in chest	*P* < 0.001 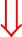	*P* < 0.001 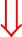	*P* < 0.05 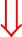	*P* < 0.05 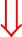
Pain in arm or shoulder	*P* < 0.001 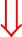	*P* < 0.001 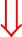	
Pain in other parts	*P* < 0.001 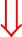	*P* < 0.001 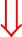	*P* < 0.05 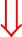	*P* < 0.05 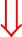
**GENERAL HEALTH STATUS (EQ-5D-5L)**				
	*P =* 0.002	-	*P =* 0.0006	*P =* 0.0004

*
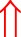
(Red), indicates significant improvement in Qol indicators; 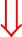
(Red), indicates significant reduction in symptoms; 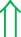
(Green), indicates significant worsening in symptoms; Qol, quality of life; EORTC QLQ-C30, European Organization for Research and Treatment of Cancer quality of life core questionnaire; QLQ-LC13, module for lung cancer; EQ-5D, EuroQol Group 5-Dimension Self-Report Questionnaire*.

### Quality Assessment

Quality of the RCTs was assessed by Jaded et al. method (42). Jaded et al.'s method is a 3 questions based method. These questions addresses whether or not the trial has reported the following three items: appropriate randomization (0–2 points); blinding (0–2 points); and withdrawals and dropouts (0–1 point). A trial achieving < 3 points was considered low quality while ≥3 points was considered high quality. All the RCTs included in this meta-analysis achieved a score of 3 points (42). Newcastle-Ottawa Scale was used to assess the quality of the retrospective studies (43).

### Statistical Analysis

Hazard ratios were extracted from the studies for time to event data (44). Hazard ratio for some outcomes was calculated from the time to event graphs in case there was no direct mention of hazard ratio in the study. Odds ratio with 95% CI were calculated for dichotomous data. Obtained Hazard ratios with 95% CI and odds ratio were pooled using the Review Manager (RevMan, V5.3) software provided by Cochrane Collaboration Tools. Heterogeneity was assessed with χ^2^ (Chi-square) and *I*^2^ statistic. >50% *I*^2^ statistic value was considered significant heterogeneity as well as a *p* < 0.05. Fixed effects model was used in case no significant heterogeneity was present otherwise a random effects model was applied.

## Author Contributions

All authors listed have made a substantial, direct and intellectual contribution to the work, and approved it for publication.

### Conflict of Interest Statement

The authors declare that the research was conducted in the absence of any commercial or financial relationships that could be construed as a potential conflict of interest.

## Abbreviations

ALK: Anaplastic lymphoma kinase
NSCLC: Non-small cell lung cancer
OS: Overall survival
ORR: Objective response rate
PFS: Progression free survival
HR: Hazard ratio
OR: Odds ratio
RCT: Randomized controlled trial
EGFR: Epidermal growth factor receptor
KRAS: Kirsten RAt Sarcoma
EML4: Echinoderm microtubule-associated protein-like 4
MET: mesenchymal-epithelial transition factor (MET)
ROS1: Proto-oncogene tyrosine-protein kinase ROS
CNS: Central nervous system
AEs: Adverse events
BBM: Baseline brain metastases
mBBM: Measurable baseline brain metastases
SE: Standard error
CI: Confidence interval
X2: Chi square test.
